# Disequilibrium in the Thioredoxin Reductase-1/Thioredoxin-1 Redox Couple Is Associated with Increased T-Cell Apoptosis in Children with Autism

**DOI:** 10.3390/metabo13020286

**Published:** 2023-02-16

**Authors:** Samiyah Alshehri, Ahmed Nadeem, Sheikh F. Ahmad, Sana S. Alqarni, Naif O. Al-Harbi, Laila Y. Al-Ayadhi, Sabry M. Attia, Saleh A. Alqarni, Saleh A. Bakheet

**Affiliations:** 1 Department of Pharmacology and Toxicology, College of Pharmacy, King Saud University, Riyadh 11451, Saudi Arabia; 2 Department of Medical Laboratory Science, College of Applied Medical Sciences, King Saud University, Riyadh 11451, Saudi Arabia; 3 Department of Physiology, College of Medicine, King Saud University, Riyadh 11451, Saudi Arabia

**Keywords:** ASD, T cells, TrxR1, Trx1, inflammation, immune system, apoptosis

## Abstract

Autism spectrum disorder (ASD) is a neuropsychiatric childhood disorder that affects social skill and language development, and is characterized by persistent stereotypic behaviors, restricted social interests, and impaired language/social skills. ASD subjects have dysregulated immune responses due to impairment in inflammatory and antioxidant signaling in immune cells, such as T cells. Thioredoxin reductase-1 (TrxR1) and thioredoxin-1 (Trx1) play a crucial role in the maintenance of redox equilibrium in several immune cells, including T cells. T-cell apoptosis plays a crucial role in the pathogenesis of several inflammatory diseases. However, it remains to be explored how the TrxR1/Trx1 redox couple affects T-cells apoptosis in ASD and typically developing control (TDC) groups. Therefore, this single-center cross-sectional study explored the expression/activity of TrxR1/Trx1, and Bcl2, 7-AAD/annexin V immunostaining in T cells of ASD (*n* = 25) and TDC (*n* = 22) groups. Further, effects of the LPS were determined on apoptosis in TDC and ASD T cells. Our data show that T cells have increased TrxR1 expression, while having decreased Trx1 expression in the ASD group. Further, TrxR enzymatic activity was also elevated in T cells of the ASD group. Furthermore, T cells of the ASD group had a decreased Bcl2 expression and an increased % of annexin V immunostaining. Treatment of T cells with LPS caused greater apoptosis in the ASD group than the TDC group, with same treatment. These data reveal that the redox couple TrxR1/Trx1 is dysregulated in T cells of ASD subjects, which is associated with decreased Bcl2 expression and increased apoptosis. This may lead to decreased survival of T cells in ASD subjects during chronic inflammation. Future studies should investigate environmental factors, such as gut dysbiosis and pollutants, that may cause abnormal immune responses in the T cells of ASD subjects due to chronic inflammation.

## 1. Introduction

Autism Spectrum Disorders (ASD) is a neuropsychiatric disorder of the childhood that affects cognition, language development and social communication, and is characterized by a wide range of behavioral problems, such as persistent stereotypic behaviors, restricted social interests, and impaired language/social skills [1,2]. ASD can significantly affect the quality of life starting from early childhood, and is a huge economic burden on the healthcare system [2]. The pathophysiology of ASD is quite complex, but is thought to be the result of interactions between genetic and environmental factors that affect the neurodevelopmental program during fetal life or early childhood. In the last two decades, there has been a gradual increase in the prevalence of ASD, with current estimates being between 1–2% [3,4,5].

The thioredoxin system consists of thioredoxin reductase (TrxR), thioredoxin (Trx), and NADPH. The redox couple TrxR/Trx is a major antioxidant system that is involved in the homeostasis of the cell. TrxR has several isoforms: TrxR1, being the cytosolic form, plays a dominant role in the detoxification of toxic-free radicals, directly or indirectly in conjunction with Trx and NADPH [6,7]. Trx1, along with TrxR1, also regulates other enzymes that need reducing equivalents in the form of electrons, such as peroxiredoxins, ribonucleotide reductases, and methionine sulfoxide reductases [8,9]. Dysregulation of the redox couple TrxR1/Trx1 may lead to deleterious effects in the form of a compromised antioxidant enzymatic capacity and dysregulated synthesis of important biomolecules, such as DNA. As a result, functions of a cell, such as proliferation capacity and detoxification of ROS, may be affected [10,11].

TrxR1/Trx1 is essential for the normal replication activity of immune cells, as it regulates ribonucleotide reductase activity, which is essential for DNA replication [6,12]. The TrxR1/Trx1 redox system also controls the apoptotic process through various mechanisms, such as maintenance of anti-apoptotic protein Bcl2. Therefore, Trx1/TrxR1 expression/activity may be required for proliferative and antiapoptotic effects of immune cells, such as T cells [7,13]. The anti-apoptotic properties of the Trx1 could be important for the survival of T cells in an inflammatory environment. The Trx1/TrxR1 expression/activity has been shown to be enhanced in various immune cells to counteract oxidative stress and apoptotic processes including T cells [14,15]. However, it has not been explored in the context of ASD.

ASD subjects have been reported to have an oxidant–antioxidant disequilibrium in both the peripheral circulatory system and the central nervous system [16,17,18]. Several enzymatic and non-enzymatic antioxidants are dysregulated in immune cells of ASD subjects. SOD, GPx, GR have been reported to be dysregulated in different immune cells, such as monocytes, neutrophils, B cells, and T cells [16,17,18]. The Trx/TrxR1 redox couple has also been reported to be dysregulated in ASD subjects, however most of these studies were carried out in the plasma/serum/blood [19,20].

Therefore, this study attempted to explore the expression/activity of TrxR1 and Trx1 in T cells of ASD children and typically developing children (TDC). Further, the effect of TLR4 activation by lipopolysaccharide (LPS) on the apoptosis of T cells in ASD/TDC subjects was determined. For this purpose, PBMCs and T cells isolated from ASD and TDC groups were utilized for the measurement of TrxR1, Trx1, Bcl2 and apoptosis. Our study showed significant differences in the Trx1/TrxR1 redox couple and apoptosis parameters in the T cells of the ASD group, as compared to the TDC group, which may affect T-cell immune responses in the ASD group.

## 2. Materials and Methods

### 2.1. Participants

This investigation included 25 children with ASD (21 males and 4 females; age: 6.90 ± 2.65 years, mean ± SD) and it was a single-center cross-sectional study. ASD children were recruited from the Autism Research and Treatment Center, Faculty of Medicine, King Saud University. Those children who had a history of metabolic, neuropsychiatric/neurological diseases (e.g., depression, phenylketonuria, seizures, tuberous sclerosis, mental retardation) and inflammatory/autoimmune diseases (psoriasis, rheumatoid arthritis) were excluded from the study. Children who met the above criteria were included in this investigation. The other group had 22 typically developing control (TDC) children (18 males and 4 females; age: 7.2 ± 2.85 years, mean ± SD), who were recruited from the Well Baby Clinic, King Khalid University Hospital, Faculty of Medicine, King Saud University, Riyadh, Saudi Arabia. TDC children who had any symptoms or history related to autoimmune/inflammatory diseases, or any language/intellectual impairment or any known genetic diseases, were excluded from the investigation. Both groups were healthy and active at the time of venipuncture, and were not on any vitamin- or immune-modifying drugs. The children’s parents/guardians provided permission for the participation in the study for their respective children. An approval from the local Ethical Committee of the Faculty of Medicine, King Saud University, Riyadh, Saudi Arabia, was obtained for this investigation. ASD and TDC children did not have any significant differences in basal growth characteristics and inflammatory/neurological disease history.

### 2.2. Study Measurements

Diagnosis of ASD in the children was established after interviewing the subjects, which was carried out by well-trained medical staff. Assessment of the subjects comprised of medical history, clinical examination, and neuropsychiatric inspection, according to the recommended guidelines for the diagnosis of autism provided in the 5th edition of the *Diagnostic and Statistical Manual of Mental Disorders* (American Psychiatric Association, 2013) [21]. Moreover, the severity of ASD symptoms were assessed according to the Childhood Autism Rating Scale (CARS), which provides a scale of 1–4 in 15 different areas (with a maximum score of 60) for each subject, as detailed before [22]. According to the CARS assessment, the ASD group was divided into two subgroups: mild–moderate ASD group (M-ASD group; *n* = 15) with a score of 30–36; severe ASD group (S-ASD group; *n* = 10) with a score of 37–60.

### 2.3. Separation of Peripheral Blood Mononuclear Cells (PBMCs)/T Cells

PBMCs from peripheral blood were separated by density gradient centrifugation, as stated earlier [23]. Briefly, venous blood was collected in an acid-citrate dextrose Vacutainer tube (BD Biosciences; Franklin Lakes, NJ, USA) through venipuncture, followed by separation of PBMCs using the Ficoll-Paque (1.077 g/mL; Sigma-Aldrich, St. Louis, MO, USA) density gradient. PBMCs were utilized to further separate T cells from other remaining immune cells (B cells, NK cells, monocytes, granulocytes, dendritic cells, platelets, etc.) using the Dynabeads™ Untouched™ Human T Cells kit (Thermofisher Scientific, Waltham, MA, USA).

### 2.4. T-Cell TrxR Activity Assay

Assessment of TrxR in T cells was evaluated by a commercial kit (Cayman Chemical, Ann Arbor, MI, USA). This kit was based on the appearance of a color produced as a result of TrxR enzymatic activity, which can be read at 405 nm spectrophotometrically. Appearance of color is directly proportional to the activity of TrxR in T cells. Results are expressed as nmol/min/mg protein.

### 2.5. Flow Cytometry

PBMCs collected from the subjects of ASD and TDC were assayed for the analysis of different proteins by flow cytometry, as detailed earlier [23,24]. PBMCs were first labeled with fluorescently coupled antibodies (FITC/PE/APC; Biolegend, San Diego, CA, USA) against an extracellular cell surface protein, i.e., CD3. This followed the normal steps of permeabilization and fixation to prepare the cells for intracellular immunostaining. Thereafter, PBMCs were labeled with fluorescently coupled (FITC/APC) antibodies against intracellular proteins, i.e., Trx1, TrxR1, and Bcl2 (Santa Cruz Biotech, Dallas, TX, USA; Biolegend, USA). PBMC samples were run in a flow cytometer (Cytomics FC500 software, Beckman Coulter, Brea, CA, USA) and 10,000 cells were acquired for the assessment of cell surface and intracellular proteins as stated before [23,24].

### 2.6. PBMC/T-Cell Culture

T cells/PBMCs isolated from the subjects of ASD and TDC were incubated under standard conditions in 24-well culture plates for apoptosis assessment, with or without LPS (0.1 µg/mL), in RPMI 1640 medium plus heat-inactivated FBS (Invitrogen, Waltham, MA, USA; Gibco, Grand Island, NY, USA), in parallel with concavalin (5 μg/mL)-mediated T-cell activation. T cells/PBMCs were kept in standard culture conditions for 24 h, followed by apoptosis assessment via flow cytometry, as written below.

### 2.7. T-Cell Apoptosis Assay

Apoptosis was determined by staining the T cells after cell culture with annexin V and 7-AAD stains, according to the protocol of the manufacturer (Biolegend, San Diego, CA, USA). Annexin V usually stains early apoptotic cells, whereas double immunostaining with annexin V and 7-AAD is indicative of late apoptotic/necrotic cells.

### 2.8. Statistical Analysis

The data were displayed as the mean ± SEM. Samples from each subject were run in duplicate for the biochemical assays listed above. Comparisons among different groups were assessed and analyzed by a one-way ANOVA (analysis of variance), followed by a multiple comparison post-hoc test, i.e., a Tukey’s test. The distribution of the data was evaluated by a Shapiro–Wilk test, which showed that the data were normally distributed. The level of statistical significance was set at *p* < 0.05. All the statistical analyses were conducted utilizing the Graph Pad Prism 9 statistical package (GraphPad Software, San Diego, CA, USA). Power analysis was conducted using the G*Power statistical software, which provided a sample size of ≤15 for each group.

## 3. Results

### 3.1. TrxR1 Expression Is Upregulated in the T Cells of ASD Subjects

**Figure 1:**
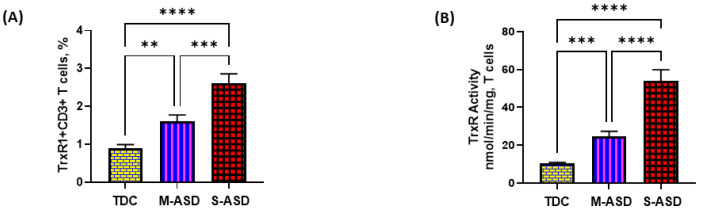

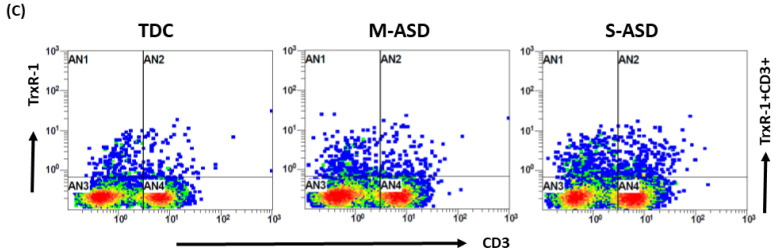
TrxR expression/activity in the T cells of the ASD and TDC groups. (**A**), TrxR1+CD3+T cells, (**B**) TrxR activity in T cells, (**C**) Flow plot displaying the double-positive immunostaining of TrxR1+CD3+T cells. PBMCs and T cells were segregated from whole blood, followed by flow cytometry and enzymatic assay, respectively. The ASD group was further divided into mild–moderate (M-ASD) and severe (S-ASD). Values are shown as the mean ± SEM, *n* = 22–25/group. ** *p* < 0.01; *** *p* < 0.001, **** *p* < 0.0001.

Our results show that TrxR1 protein expression is elevated in ASD T cells as compared to TDC T cells. This was reflected by the double-positive immunostaining of the TrxR1+CD3+T cells (Figure 1A,C). Further, when categorized according to severity, CD3+T cells from the S-ASD group had a further elevation in TrxR1 expression as compared to the M-ASD group (Figure 1A,C). Furthermore, TrxR activity was evaluated in the T cells of the ASD and TDC groups. Our results show that TrxR activity was also elevated in ASD T cells as compared to TDC T cells (Figure 1B). When categorized according to severity, T cells from the S-ASD group had a further elevation in TrxR enzymatic activity compared to the M-ASD group (Figure 1B). These results exhibit that TrxR1 expression/activity are elevated in T cells of ASD children, which may be due to the dysregulated oxidant–antioxidant balance.

### 3.2. Trx1 Expression Is Downregulated in the T Cells of ASD Subjects

**Figure 2:**
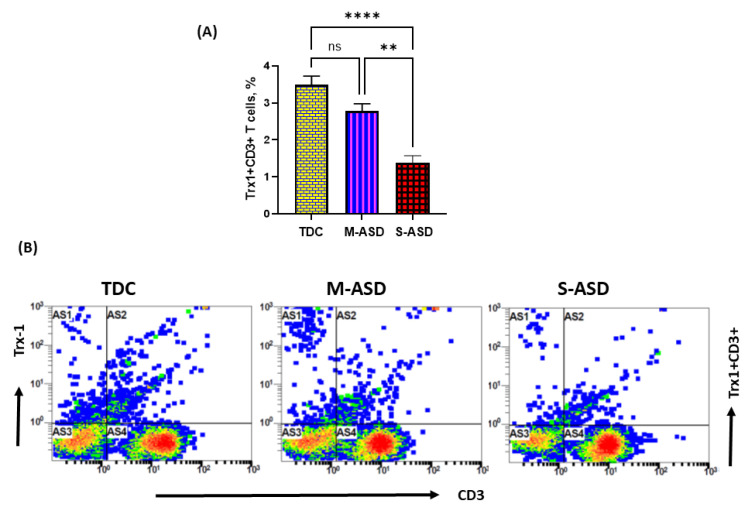
Trx1 expression in the T cells of the ASD and TDC groups. (**A**), Trx1+CD3+T cells, (**B**) Flow plot displaying the double-positive immunostaining of Trx1+CD3+T cells. PBMCs were segregated from whole blood, followed by flow cytometry. The ASD group was further divided into mild–moderate (M-ASD) and severe (S-ASD). Values are shown as the mean ± SEM, *n* = 22–25/group. ** *p* < 0.01; **** *p* < 0.0001.

Our results show that Trx1 protein expression is decreased in M-ASD CD3+T cells compared to TDC CD3+T cells, however this was not significant (Figure 2A,B). Further, when categorized according to severity, CD3+T cells from the S-ASD group had a further decrease in Trx1 expression compared to the M-ASD group and TDC group (Figure 2A,B). This was evident from the decreased double-positive immunostaining of Trx1+CD3+T cells. These results display that Trx1 expression is decreased in the T cells of ASD children, which may be due to increased oxidative stress.

### 3.3. T Cells from ASD Have Decreased Bcl2 and Increased Apoptosis

**Figure 3:**
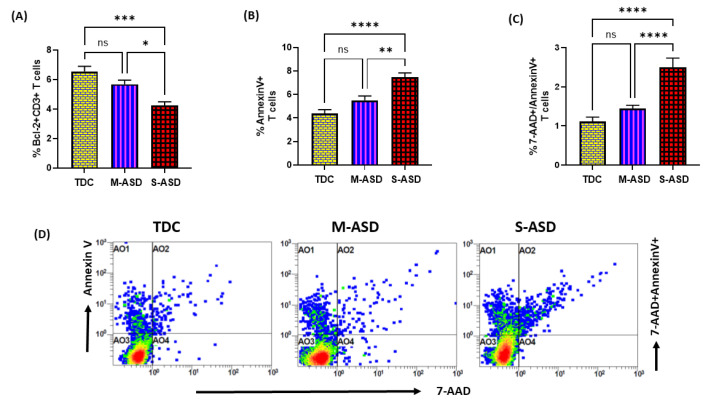
Bcl2 expression and apoptosis in the T cells of the ASD and TDC groups. (**A**) Bcl-2+ T cells, (**B**) annexin V+ T cells, (**C**) annexin V+/7-AAD+ T cells, and (**D**) Flow plot displaying the double-positive immunostaining of annexin V+/7-AAD+T. PBMCs were segregated from whole blood, followed by flow cytometry. The ASD group is further divided into mild–moderate (M-ASD) and severe (S-ASD). Values are shown as the mean ± SEM, *n* = 22–25/group. * *p* < 0.05; ** *p* < 0.01; *** *p* < 0.001; **** *p* < 0.0001.

Next, we wanted to explore whether the dysregulation in the TrxR1/Trx1 redox couple had any effect on anti-apoptotic protein Bcl2 and apoptosis in the T cells of the ASD and TDC groups. Our data show that Bcl2 protein expression is decreased in the ASD CD3+T cells as compared to the TDC CD3+T cells. This was evident from the double-positive immunostaining of Bcl2+CD3+T cells (Figure 3A). Further, when categorized according to severity, CD3+T cells from the S-ASD group had a further decrease in Bcl2 expression as compared to the M-ASD group and TDC group (Figure 3A). Furthermore, annexin V+ single immunostaining and 7-AAD+/annexin V+ double immunostaining were greater in the concanavalin-stimulated T cells of the S-ASD group as compared to the M-ASD and TDC groups (Figure 3B–D). These results display that a decreased expression of anti-apoptotic protein, Bcl2, may be responsible for increased apoptosis in the T cells of ASD children.

### 3.4. ASD T Cells Have Increased Apoptosis in Response to TLR4 Activation

**Figure 4:**
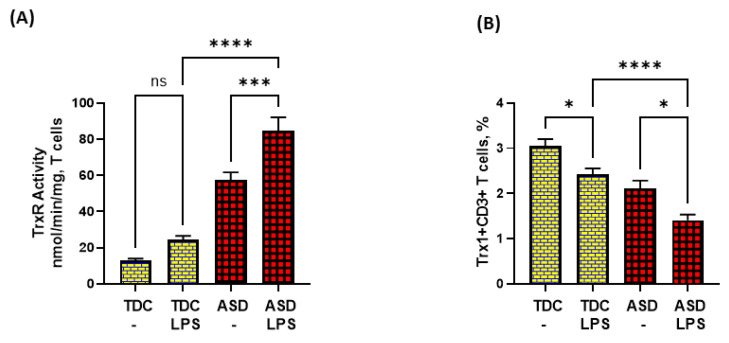
TrxR activity and Trx1 expression in the T cells of the ASD and TDC groups in response to LPS. (**A**) TrxR activity, and (**B**) % Trx1+CD3+ T cells. PBMCs and T cells were segregated from whole blood, followed by treatment with LPS, flow cytometry and enzymatic assays, respectively. Values are shown as the mean ± SEM, *n* = 15/group. * *p* < 0.05; *** *p* < 0.001; **** *p* < 0.0001.

Lastly, we attempted to explore if dysregulated TrxR1/Trx1 and Bcl-2 signaling in ASD T cells had any effect on T-cell apoptosis in response to TLR4 activation. Our data show that LPS caused enhanced TrxR activity in the ASD group, but failed to elevate Trx1 expression in T cells. On the contrary, LPS caused further downregulation of Trx1 expression in T cells (Figure 4A,B).

**Figure 5:**
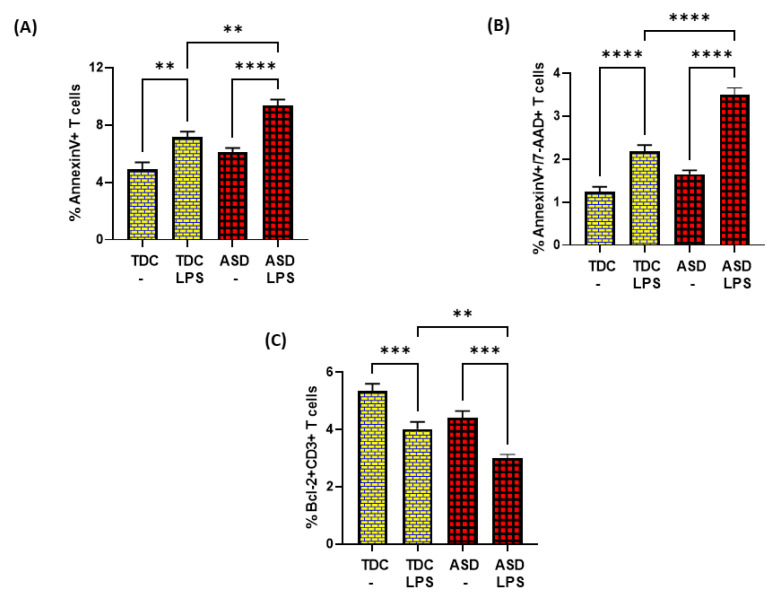
Bcl2 expression and apoptosis in the T cells of the ASD and TDC groups in response to LPS. (**A**) Annexin V+ T cells, (**B**) Annexin V+/7-AAD+ T cells, and (**C**) Bcl-2+ T cells. PBMCs were segregated from whole followed and treated with LPS, followed by flow cytometry. Values are shown as the mean ± SEM, *n* = 15/group. ** *p* < 0.01; *** *p* < 0.001; **** *p* < 0.0001.

Further, LPS caused elevated apoptosis in both ASD and TDC groups, as depicted by annexin V+ single immunostaining and 7-AAD+/annexin V+ double immunostaining in T cells (Figure 5A,B). However, the T cells of ASD had a greater % of annexin V+ immunostaining and 7-AAD+/annexin V+ immunostaining in the ASD group than the T cells of the TDC group in response to LPS (Figure 5A,B). Lastly, the effects of LPS were assessed on Bcl-2 expression in the T cells of both groups. Our data show that LPS caused a significant decrease in Bcl-2 expression in the T cells of the ASD and TDC groups, as depicted by Bcl-2+CD3+ T cells (Figure 5C). However, the T cells of ASD had a greater % decrease in Bcl-2+ T cells than the T cells of the TDC group in response to LPS (Figure 5C). This suggests that during chronic inflammation, ASD T cells may be undergoing more apoptosis than TDC T cells.

## 4. Discussion

The etiology of ASD is complex and involves several factors, which include genetic and environmental factors. A combination of these two factors affects the metabolic, immune, and epigenetic programming of the neuronal system, leading to dysfunction in social communication skills and language impairment, two key features of ASD [1,25]. Immune system dysfunction is quiet in subjects of ASD, which may be a consequence of environmental factors during early childhood or epigenetic changes inherited from the mother [26,27]. T cells are known to be important for the overall modulation of immune function, and antioxidants are known to play a significant role in the normal functioning of T cells; therefore, we evaluated an important enzymatic antioxidant, TrxR1, and its substrate Trx1, in the T cells of ASD and TDC subjects. The thioredoxin system (TrxR-1 and Trx-1) acts as a crucial regulator of cellular redox. TrxR1 is predominantly located in the cytoplasm and is one of the family members of the enzyme class nucleotide disulfide oxidoreductase. TrxR1 takes care of several cellular functions, which include antioxidant defense through the reduction in oxidative/nitrosative stress [7,8,28]. Our study shows that the TrxR1 and Trx1 redox couple is dysregulated in the T cells of ASD children compared to the TDC group. Further, T-cell apoptosis was increased, and anti-apoptotic protein Bcl-2 was decreased in the ASD group.

Expression/activity of TrxR1 has been shown to be elevated in the systemic circulation of ASD subjects compared to the TDC group. Similarly, Trx1 has also been shown to be enhanced in the serum/blood of ASD subjects [19,20,29]. This was associated with increased oxidative stress in ASD subjects. This suggests that upregulated Trx1 could be a response to increased oxidants in the systemic circulation of ASD subjects [16,17,20,23,24,30]. However, none of the studies measured their expression of T cells in ASD subjects. Our study shows, for the first time, that TrxR1 is upregulated, while Trx1 is downregulated in ASD T cells. Activity of TrxR1 may be increased in the T cells of ASD to counterbalance the decrease in expression of its substrate, Trx1.

Trx1, a key redox gatekeeper, is ubiquitously present in the cytosol of mammalian cells and is involved in the maintenance of redox balance through interaction with other molecules, such as NADPH. Trx1 possesses the disulfide bond (Cys32–Cys35) and is a natural substrate for the enzyme, TrxR1. When reduced, the disulfide bond (Cys32–Cys35) in Trx1 causes a reduction of other cellular targets, such as lipid hydroperoxides, hydrogen peroxide, and dehydroascorbate, peroxiredoxins. The Trx1/TrxR1 redox couple also regulates the activity of ribonucleotide reductase, which is required for proper cell proliferation [6,7,8,28]. In addition, it also has other redox properties which control several cellular functions of the cells. A decrease in Trx1, with increasing disease severity in the T cells of the ASD group, could be due to the extracellular release of Trx1, as immune cells have been shown to have this phenomenon [8,28,31]. Trx1 also regulates apoptotic signals, such as Bcl2, within the cells, through a modulation of kinases and transcription factors, such as NFkB, AP-1, ASK-1, and the glucocorticoid receptor [8,14,28,32,33,34].

When T cells respond to their cognate antigens, they get activated and begin proliferating, which is followed by apoptosis of activated T cells, to regulate immune responses [10,15]. Our findings illustrate that decreased levels of Trx1 may lead to increased apoptosis of T cells due to a decreased expression of anti-apoptotic protein, Bcl-2. We propose that the maintenance of Bcl-2 expression in TDC T cells is due to the normal activity of the Trx1/TrxR1 redox couple, whereas downregulation of Bcl-2 in ASD T cells could be due to the dysregulation in this redox couple. It has been shown previously that Bcl-2 overexpression causes a delay in T-cell death, while Bcl-2 deficiency causes reduced T-cell survival. This suggests that Bcl2 is a crucial survival signal in the life of T cells [35].

We also found that the T cells of the ASD group had greater LPS-induced apoptosis, which might be due to lower levels of Bcl2 protein expression. Bcl-2 protein in T cells of ASD group was downregulated more than TDC group in response to LPS. This mechanism may be responsible for increased apoptosis in response to LPS in T cells of ASD group. It has been shown previously that Bcl-2 overexpression can protect cells from apoptosis resulting from high oxidative/nitrative stress [34,36,37,38]. Low levels of Trx1 may fail to inhibit LPS-induced downregulation of Bcl2 in the ASD group. Thus, a dysregulated expression of Trx1/TrxR1 may permit increased T-cell apoptosis in ASD T cells compared to TDC T cells. Thus, it is likely that the dysregulated expression of Trx1/TrxR1 in the T cells of the ASD group may be detrimental, as it would lead to an increased death of T cells during systemic inflammation caused by bacterial products, such as LPS.

Enhanced periods of systemic inflammation may be caused by bacterial products which can activate TLR4. It has been reported previously that ASD subjects have a dysregulated gut metabolism, which causes increased permeability of the gut and blood-brain barrier, allowing for the translocation of bacterial products from the gut to the systemic circulation and brain [39,40]. As a result, bacterial products with a potential to activate TLR4 on T cells have been identified in the systemic circulation of ASD subjects [41,42].

It has been shown earlier that inhibition of the TrxR1/Trx1 system by beta-sitosterol/Parthenolide causes apoptosis through upregulation of oxidative stress in A549 cells or HeLa cells [43,44]. Other studies showed that higher Trx expression was negatively associated with oxidative-stress-induced apoptosis in adoptively transferred T cells encountered in the tumor microenvironment [14]. Trx has also been shown to enhance survival of different immune cells through the maintenance of Bcl2 expression [8,32]. It has been reported that when Trx is externally added to apoptotic cerebellar granule cell cultures, a decreased cell death is observed, indicating that the Trx/TrxR system is required for cell survival [45]. Trx1 expression has been shown to be elevated in the synovial tissue of rheumatoid arthritis patients. Trx1 overexpression has also been associated with Bcl2 expression and decreased apoptosis in fibroblasts of rheumatoid arthritis patients. On the other hand, knocking down Trx1 has been shown to downregulate Bcl2 and increase apoptosis in fibroblast-like synoviocytes [34].

Apoptosis is a critical step in the life of immune cells, such as T cells. It may have both detrimental and beneficial effects, depending on the context. During immunodeficiency disorders, apoptosis of T cells may be detrimental, as they are required to protect the body against foreign pathogens, whereas in an autoimmune disease, T-cell survival may be detrimental as it could lead to exaggerated immune responses [15,46]. It has been shown in ASD subjects that they are likely to have an increased incidence of infections [47,48]. This could be due to the increased apoptosis of T cells, as they are heavily involved in controlling bacterial/viral infections. Increased apoptosis of T cells in ASD subjects may be specifically encountered in situations where bacterial products are chronically elevated in the systemic circulation [41,42]. These observations may be used to predict T-cell immune responses in the ASD group. A T-cell apoptosis assay may be developed as a potential biomarker to test the efficacy of different treatment strategies in vitro, before undertaking a clinical trial in the ASD group.

This study has some limitations. Firstly, the male to female ratio was high due to the inherent nature of the disorder, therefore it was difficult to recruit the same number of subjects for both genders. Secondly, it would be interesting to segregate T-cell responses into CD4 and CD8 subgroups, to better understand cytotoxic and helper T-cell responses, respectively; however, this was not possible due to the limited sample volume.

Overall, our data suggest that the redox couple Trx1/TrxR1 is dysregulated in the T cells of the ASD group, which is associated with decreased anti-apoptotic protein Bcl-2. In the context of chronic systemic inflammation experienced by ASD subjects, this could lead to an increased death of T cells, which may affect normal immune responses. However, more research needs to be undertaken to explore the real significance of the TrxR1/Trx1 redox couple in the context of ASD pathogenesis and immune dysregulation.

## Data Availability

The authors confirm that all data underlying the findings are fully available without restriction. All relevant data are within the paper.
